# Myosin VI in the nucleus of neurosecretory PC12 cells: Stimulation-dependent nuclear translocation and interaction with nuclear proteins^*^

**DOI:** 10.1080/19491034.2017.1421881

**Published:** 2018-01-30

**Authors:** Lukasz Majewski, Jolanta Nowak, Magdalena Sobczak, Olena Karatsai, Serhiy Havrylov, Robert Lenartowski, Malgorzata Suszek, Marta Lenartowska, Maria Jolanta Redowicz

**Affiliations:** a Laboratory of Molecular Basis of Cell Motility, Department of Biochemistry, Nencki Institute of Experimental Biology, Polish Academy of Sciences, Warsaw, Poland; b Laboratory of Isotope and Instrumental Analysis, Department of Cellular and Molecular Biology, Faculty of Biology and Environmental Protection, Nicolaus Copernicus University in Torun, Torun, Poland; c Laboratory of Developmental Biology, Department of Cellular and Molecular Biology, Faculty of Biology and Environmental Protection, Nicolaus Copernicus University in Torun, Torun, Poland

**Keywords:** hnRNPU, histone, myosin VI, nucleolin, nucleus, PC12 cells, PML bodies, speckles, transcription

## Abstract

Myosin VI (MVI) is a unique actin-based motor protein moving towards the minus end of actin filaments, in the opposite direction than other known myosins. Besides well described functions of MVI in endocytosis and maintenance of Golgi apparatus, there are few reports showing its involvement in transcription. We previously demonstrated that in neurosecretory PC12 cells MVI was present in the cytoplasm and nucleus, and its depletion caused substantial inhibition of cell migration and proliferation. Here, we show an increase in nuclear localization of MVI upon cell stimulation, and identification of potential nuclear localization (NLS) and nuclear export (NES) signals within MVI heavy chain. These signals seem to be functional as the MVI nuclear presence was affected by the inhibitors of nuclear import (ivermectin) and export (leptomycin B). In nuclei of stimulated cells, MVI colocalized with active RNA polymerase II, BrUTP-containing transcription sites and transcription factor SP1 as well as SC35 and PML proteins, markers of nuclear speckles and PML bodies, respectively. Mass spectrometry analysis of samples of a GST-pull-down assay with the MVI tail domain as a “bait” identified several new potential MVI binding partners. Among them are proteins involved in transcription and post-transcriptional processes. We confirmed interaction of MVI with heterogeneous nuclear ribonucleoprotein U (hnRNPU) and nucleolin, proteins involved in pre-mRNA binding and transport, and nucleolar function, respectively. Our data provide an insight into mechanisms of involvement of MVI in nuclear processes *via* interaction with nuclear proteins and support a notion for important role(s) for MVI in gene expression.

## Introduction

Reports from almost two decades have definitely confirmed the functional presence of actin and actin-based molecular motors within the nucleus [1–3]. However, understanding of molecular functions of these cytoskeletal components in nuclear processes is still very limited.

Myosins, the structurally and functionally diverse actin-dependent motor proteins, are expressed in all eukaryotic cells and are involved in a variety of cellular processes associated with the actin-based cytoskeleton, such as muscle contraction, cell motility, vesicle-mediated transport, endo- and exocytosis, cell-matrix adhesion, and gene transcription [4,5]. They are composed of one or two heavy chains and up to seven light chains (in most cases calmodulin) per one heavy chain, dependent on a myosin type. Heavy chains consist of the N-terminal motor domain, the so-called neck or IQ domain, where light chains are non-covalently bound, and the most diverse C-terminal tail domain, responsible for cargo binding and/or dimerization [4,5]. The classic two-headed myosins are called conventional (classified as class II), while all other myosins, referred to as unconventional myosins, are grouped into at least 30 different families [6].

So far, several unconventional myosins have been found in the nucleus: nuclear isoform of myosin IC (NMIC isoforms b and c), II (non-muscle isoform), myosins VA and VB, VI, XVIB, and XVIIIA and XVIIIB [1,3]. They are thought to interact with nuclear actin and participate in intranuclear trafficking, DNA replication and repair, as well as in transcription.

Myosin VI (MVI) is the only known myosin that moves towards the minus (pointed) end of actin filaments [7]. Its heavy chain has a molecular weight of 140-kDa with C-terminal part of the tail forming a globular domain involved in cargo binding *via* interaction with the binding partners [8–12]. The inverse MVI movement, resulting from difference in the structure of the converter and neck regions implies its involvement in distinct cellular functions, as compared to other myosins [9,13]. Mammalian cells express four splice variants of MVI differing by the presence of insertions within the tail domain, which seem to determine the MVI distribution and functions [14–16]. Besides interaction of MVI with its numerous partners, it was shown that the positively charged tail region could bind to PIP_2_-containing liposomes [17]. These interactions are believed to define role(s) of MVI in particular cell types or tissues. Mutations within the MVI gene are associated with hearing loss in mice and humans [18]. Several other defects were also reported in different tissues and cell lines derived from the MVI knock-out Snell's waltzer mice [19–22]. Noteworthy, MVI was shown to be overexpressed in ovarian and prostate cancers, and inhibition of its expression in tumor cells significantly attenuated cancer cell invasiveness *in vitro* [23,24]. Data collected so far indicate that MVI plays important roles in endocytic trafficking as well as in cell motility, and it may act as a transporting motor or an anchor linking vesicles and/or plasma membrane proteins to the actin cytoskeleton, thus regulating organization of the cytoskeleton [9,11].

In the nucleus, MVI was found in chromatin-free regions, where it was associated with the RNA polymerase II transcription machinery indicating its potential involvement in gene transcription [25–27]. This notion was also confirmed by the studies demonstrating involvement of MVI in the p53-dependent pro-survival pathway [25,28] and suggesting its modulatory role in androgen-dependent gene expression [29]. Recently, it has been shown that this molecular motor regulates gene pairing and transcriptional pause release in T cells [30].

In neurosecretory PC12 cells, MVI is associated with the chromaffin granules, synaptic vesicles, Golgi apparatus, endoplasmic reticulum, early endosomes and clathrin-coated vesicles, and is also present within the nucleus [26]. We showed important roles for MVI in cell migration and proliferation, but not in catecholamine secretion [31]. Moreover, we demonstrated that interaction of MVI with the newly identified partner, DOCK7, was crucial for the NGF-stimulated outgrowth formation [32,33]. In the present study, we demonstrate for the first time that upon PC12 cell stimulation MVI translocates to the nucleus, where it colocalizes not only with transcriptionally active regions, but also with PML bodies and speckles. Moreover, we have identified several MVI potential protein partners that are involved in the processes associated with gene expression and intranuclear transport. Among them is heterogeneous nuclear ribonucleoprotein U (hnRNPU), a member of the complex involved in a pre-mRNA binding and transport. We believe that interaction with MVI nuclear partners might underlie the mechanism of involvement of MVI in nuclear functions.

## Results

Our observations that MVI is present within the nuclei of rat pheochromocytoma PC12 cells and in the primary cultures of bovine adrenal medulla chromaffin cells [26] as well as its importance for cell proliferation [31] and gene transcription [27] urged us to pursue for mechanisms of involvement of this molecular motor in nuclear processes.

### Stimulation-dependent nuclear translocation of MVI

**Figure 1.**
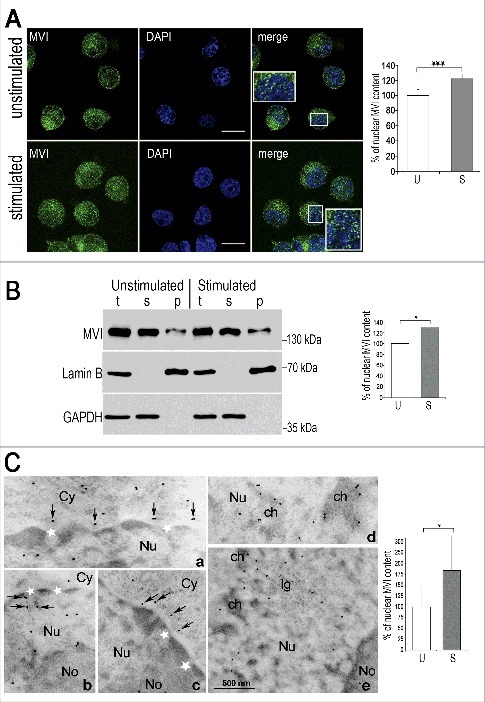
Nuclear localization of MVI is enhanced upon stimulation. A, left panel, MVI distribution in unstimulated cells and cells stimulated with 56 mM KCl for 5 min. MVI, in green, and nuclear chromatin visualized with DAPI, in blue. Insets, 2.5 x magnifications of the marked regions. Bars: 10 μm. The images are 0.4-μm sections of cell center attained with Leica confocal microscope. Right panel, quantification of MVI-associated fluorescence in the nuclei of unstimulated (U) and 5-min stimulated cells (S) using Leica software. Forty cells for each of the experimental conditions were evaluated. The values (means±SEM) represent the number of fluorescent signals in the nucleus expressed as percentage, where 100% is the mean fluorescence of unstimulated cells. B, left panel, immunoblot analysis. MVI was detected with MVI antibody in unstimulated and stimulated cells. Bands detected with anti-lamin B and anti-GAPDH antibodies serve as the internal loading control and marker of the fraction purity. Densitometric analysis (right panel) of MVI content in nuclear fraction with respect to relevant load controls is based on quantification of four blots from four independent experiments where 100% corresponds to the amount of the mean MVI signal (±SEM) in unstimulated cells. t, total homogenates; s, cytoplasmic fraction, and p, nuclear fraction. * and ***, statistical relevance according to t-Student's test *p*<0.05 and *p*<0.001, respectively. C, Immunogold technique confirms the presence of MVI in the nucleus. a-c, MVI-associated gold particles (marked with arrows) are localized near to the nuclear envelope in the vicinity of nuclear pores (white stars), both on the cytoplasmic (*Cy*) and the nucleoplasmic (*Nu*) sides, as well as in the nucleolus (*No*), independently whether the cells were (c) or were not (a and b) stimulated. Both in unstimulated (d) and stimulated (e) cells MVI-associated gold particles are visible in the edges of dense chromatin (*ch*). In stimulated cells (e), gold traces were also labeled the interchromatin space and the interchromatin granules (*Ig*). Bar, 500 nm. Right panel, quantification of the gold particles in the nuclei before (U) and after stimulation with 56 mM KCl (S) where 100% corresponds to the amount of particles (±SD) in unstimulated cells. *, statistical relevance p<0.05.

A closer examination of the MVI staining in the nucleus revealed its localization at various punctate structures located mostly at chromatin-free regions and at the cytoplasm-nucleus border (Figure 1A, insets). Moreover, an increase in the MVI-associated fluorescence was observed in the nuclei upon cell stimulation with 56 mM KCl, indicating possible translocation of MVI through the nuclear pore. The quantitative analysis of the fluorescence intensities within the cytoplasm and the nucleus before and after stimulation of cells with KCl (Figure 1A, right panel) revealed an approximately 25% increase of MVI in the nuclei of the cells upon 5-min stimulation. These observations were confirmed by quantitative immunoblot analysis of endogenous MVI content in the crude nuclear fraction before and after stimulation that demonstrated ∼30% increase of MVI in the nuclear fraction of stimulated cells (Figure 1B).

A similar observation was made with the use of the immunogold technique (Figure 1C). Gold particles associated with MVI were present both in the cytoplasm (see also Supplementary Figure 1A) and nucleoplasm as well as in the vicinity of nuclear pores, both on the cytoplasmic and nucleoplasmic sides (Figure 1C, a-c), where they were associated with fibrous electron dense material. In the nucleoplasm of unstimulated cells, MVI was present at the edges of condensed chromatin (Figure 1C, d) while in stimulated cells it was visible within de-condensed chromatin and interchromatin granules (Figure 1C, e). The number of MVI-associated gold particles in the nucleus was elevated by about 80% upon stimulation with respect to unstimulated cells (Figure 1C, right panel) thus confirming the stimulation-dependent translocation of MVI to the nucleus.

### Effect of inhibitors of nuclear import and export on MVI nuclear translocation

With Psort II [34] bioinformatic tool, we were able to identify six potential monopartite and one bipartite nuclear localization signal (NLS) regions, confined mainly to the tail of MVI heavy chain (Table 1). However, it was Vreugde et al. (2006) who first reported the presence of the NLS regions but without details about their location within the MVI heavy chain [27]. Also, with NetNES [35] bioinformatic tool we found a region flanking leucine 1014 in MVI tail that could be a potential nuclear export sequence (NES; Table 1). One NLS signal is located in the motor domain, one in the IQ motif and five in the tail, among them two in the globular tail domain. The bipartite NLS contains the RRL motif, involved in electrostatic interaction with the partners, and a positively charged region (WKSKNKKRN), involved in PIP_2_ binding [17]. It is noteworthy that the RRL motif is functional only in the MVI isoforms without the large insert which was shown to spatially mask this interaction site [36]. Thus, there are structural grounds for the MVI shuttling between the cytoplasm and the nucleus.

**Table 1.** Putative NLS and NES regions within the rat MVI heavy chain.

Putative Sites	Amino acid residues predicted by bioinformatic tools	Location within the MVI heavy chain (residues/domains)
NLS – nuclear localization signal	PRKSKLA	559-566, motor domain
	RRHK	831–834, IQ motif
	RKRR	937–940, putative coiled-coil domain
	RRRK	946–949, putative coiled-coil domain
	RKKR	971–974, putative coiled-coil domain
	RRLKVYHAWKSKNKKRN	1116–1132, globular tail domain
	PQNKKKG	1185–1191, globular tail domain
NES – nuclear export sequence	LALRI	1012–1016, putative coiled-coil domain

The amino acid residue numbering according to sequence of rat MVI gene *(Myo6)* deposited in UniProtKB database as D4A5I9_RAT.

**Figure 2.**
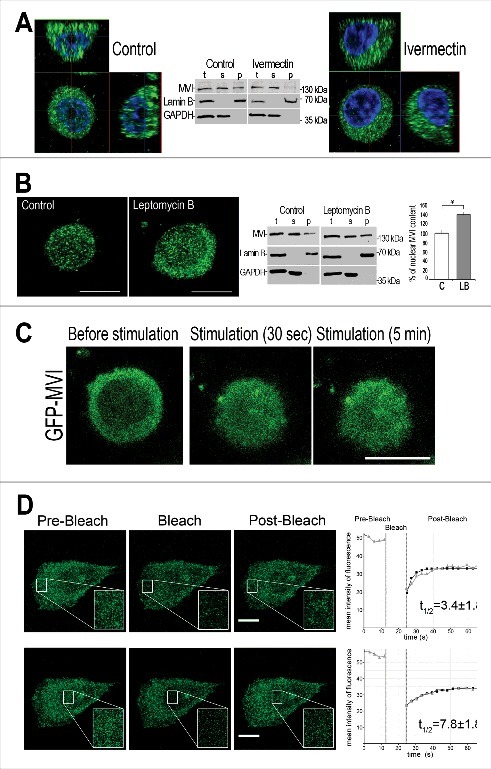
MVI shuttling between the cytoplasm and nucleus. MVI was detected with anti-MVI antibody (in A and B) or by green-fluorescence associated with GFP-MVI (in C and D). A, effect of inhibitor of nuclear import on MVI nuclear distribution. Z sections of the cells untreated (left panel) and treated for 4 hours with 25 μM ivermectin (right panel) prior to 5-min stimulation. Control cells were incubated in the same conditions as treated cells but in the presence of DMSO, in which ivermectin was dissolved. Chromatin visualized in blue with DAPI. B, effect of inhibitor of nuclear export on MVI nuclear distribution. Cell centers of the cells untreated (left panel) and treated for 3 hours with 20 nM leptomycin B (middle panel) prior to 5-min stimulation. Right panel, quantification of MVI-associated fluorescence performed as described in Figure 1A. The values (means±SEM) represent the number of fluorescent signals in the nucleus expressed as percentage, where 100% is the mean fluorescence of unstimulated cells. *, statistical relevance according to t-Student's test was *p*<0.05. Middle panels in A and B, immunoblot analysis of control cells and cells treated for 4 hours with 25 μM ivermectin or 20 nM leptomycin B, respectively. MVI was detected with MVI antibody and bands detected with anti-lamin B and anti-GAPDH antibodies serve as the internal loading control and marker of the fraction purity. t, total homogenates; s, cytoplasmic fraction, and p, nuclear fraction. C, visualization of translocation of GFP-fused MVI towards the nucleus. Cells expressing GFP-MVI were recorded *in vivo* (using time-lapse filming) before stimulation and up to 8 min after stimulation. Other details as described in Material and Methods. D, Analysis of MVI turnover using FRAP technique. Time-lapse images of cells expressing GFP-MVI before (pre-bleach) and after photobleaching (post-bleach). The regions within the cytoplasm (upper panels) and the nucleus (lower panels) were subjected to the bleaching and recovery of the fluorescence was recorded at 5-s intervals. Insets, about 3x magnification of marked regions of interest (ROI). Far right panels, plots of relative fluorescence intensity of GFP-MVI before and after photobleaching. These plots are representative for three independent experiments. Gray triangles, experimental data, black squares, the FRAP fitted single exponential curves. Other details as described in Material and Methods. Bars in A-D, 10 μm.

To check whether MVI translocation to the nucleus is dependent on nuclear import and export mechanisms, we examined the effects of ivermectin [37] and leptomycin B [38], inhibitors of nuclear import and export, respectively (Figure 2A and B; Supplementary Figure 1B and C).

In the stimulated cells incubated with 25 μM ivermectin, MVI-associated fluorescence was practically not visible in the nucleoplasm when compared to control untreated cells (Figure 2A, Supplementary Figure 1B), suggesting inhibition of MVI translocation into the nucleus. Contrary to that, in the presence of leptomycin B the amount of MVI in the nucleus of stimulated cells was significantly increased (by ∼50%) with the respect to the nuclei of untreated cells (Figure 2B, Supplementary Figure 1C). The fluorescence analyses of immunostained cells was confirmed by the cell fractionation followed by immunoblotting with anti-MVI antibody (Figure 2A and 2B, middle panels).

### Dynamics of MVI nuclear translocation

By means of time-lapse microscopy, we were also able to visualize stimulation-dependent translocation of GFP-tagged MVI protein (GFP-MVI; the isoform without a large insert that is predominant in PC12 cells [31]) to the nucleus (Figure 2C). GFP-MVI accumulated in the nuclei as early as 30 s after cell stimulation and accumulation persisted throughout the duration of the experiment (up to 8 min).

To further analyze the dynamics of nuclear and cytoplasmic pools of MVI, we performed FRAP experiments using unstimulated PC12 cells overexpressing GFP-MVI (Figure 2D). MVI-associated fluorescence was photobleached in two separate regions, in the cytoplasm and nucleus, and fluorescence recovery was then observed. In both regions, most of the fluorescence recovered within several seconds, indicating fast translocation of GFP-MVI to the bleached regions. As expected, recovery within the cytoplasm was faster than in the nucleus with the respective t_1/2_ values of 3.4±1.8 s and 7.8±1.8 s.

### Stimulation-dependent MVI nuclear translocation is associated with an increase in transcriptional activity

**Figure 3.**
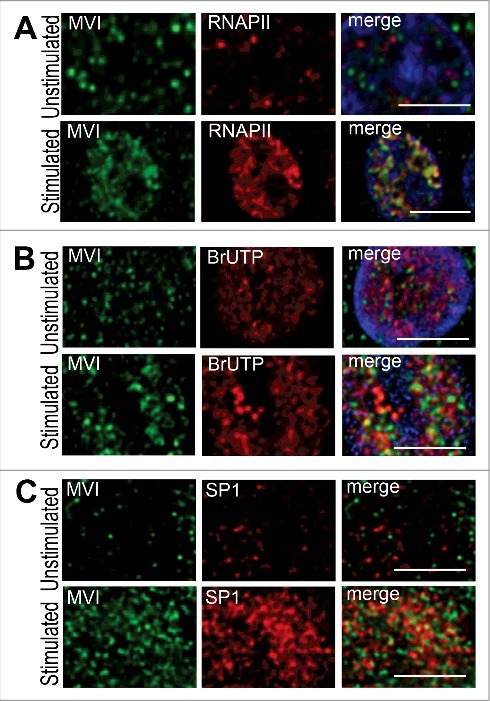
MVI upon KCl-evoked stimulation localizes within transcriptionally active regions. A, colocalization of MVI (in green) and active RNAPII (in red) is visible upon stimulation. B, colocalization of MVI (in green) with transcriptionally active sites (in red, visualized by BrUTP incorporation) is more pronounced after stimulation. C, colocalization of MVI (in green) and transcription factor SP1 (in red) is visible after stimulation. Nuclear chromatin was visualized in A and B with ToPro3 dye (in blue). Where indicated, cells were stimulated for 5 min with 56 mM KCl. These are confocal sections (0.4 μm) of the cell centers. Bars, 5 μm.

The observation that KCl stimulation evoked an increase in the content of MVI in the nucleus together with the previous report [27] that MVI is involved in transcription in HeLa cells prompted us to test whether stimulation-dependent nuclear translocation of MVI could be associated with the enhancement of transcriptional activity of PC12 cells (Figure 3).

As shown in Figures 3A and Supplementary Figure 2A, in unstimulated cells the fluorescence intensity corresponding to active RNAPII (antibody detecting the phosphorylated serine in the amino acid 5 position of the C-terminal CTD domain of the protein) was very weak. Upon 5-min stimulation the fluorescence intensity was much stronger, indicating stimulation-activated transcription. In quiescent cells there was barely any MVI and RNAPII colocalization, but it was substantially increased upon stimulation. As demonstrated in Supplementary Figure 2A and B, the value of Pearson's coefficient was 0.19 for unstimulated and 0.87 for stimulated cells. Similar observation was made when transcriptional activity was assessed by means of BrUTP incorporation (Figure 3B and Supplementary Figure 3). As expected, BrUTP-associated signal was more intense in the stimulated cells and so was its colocalization with MVI. Also, we noticed that colocalization of MVI with transcription factor SP1 was enhanced in stimulated cells (Figure 3C and Supplementary Figure 4).

**Figure 4.**
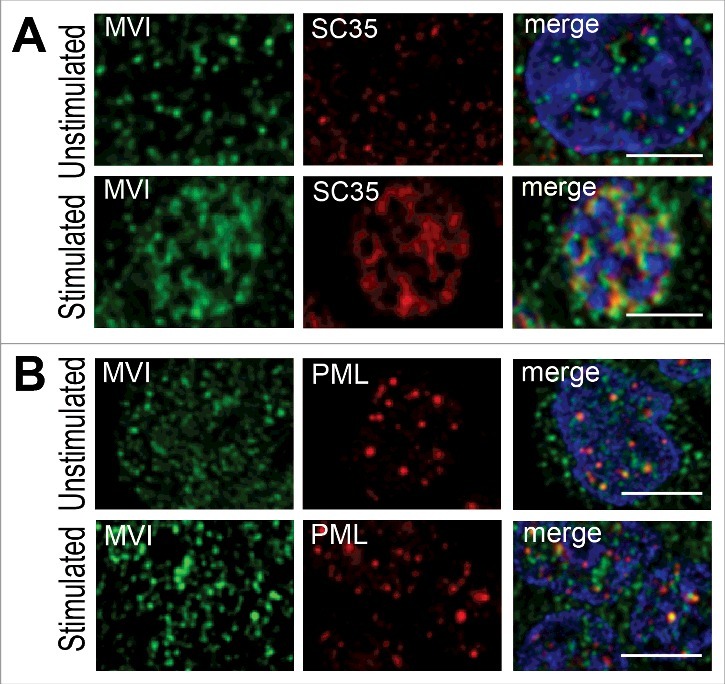
MVI localization within nuclear bodies. A, upon KCl-evoked stimulation MVI (in green) colocalizes with nuclear speckle protein SC35 (in red). B, colocalization of MVI (in green) with PML bodies (in red) is not dependent on stimulation. Where indicated, cells were stimulated for 5 min with 56 mM KCl. Nuclear chromatin was visualized with ToPro3 dye (in blue). These are confocal sections (0.4 μm) of the cell centers. Bars, 5 μm.

### MVI colocalization with the nuclear structures increases upon cell stimulation

To further characterize MVI localization in the nucleus, we performed co-stainings for endogenous MVI and markers of nuclear speckles (SC35), structures involved in pre-mRNA processing, and PML bodies (PML protein), structures involved in several processes such as gene transcription, chromatin reconstruction and DNA repair [39] (Figure 4 and Supplementary Figures 5 and 6).

**Figure 5.**
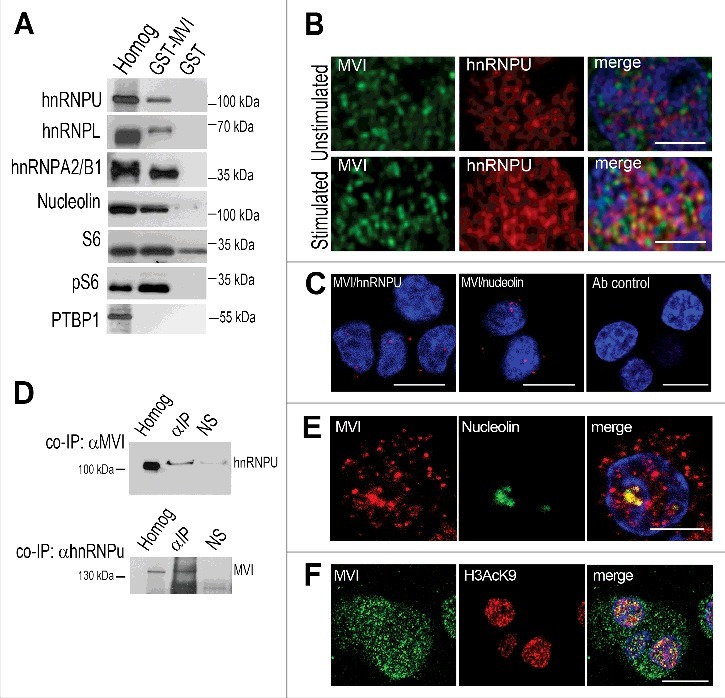
Probing MVI interaction with its putative partners. A, immunoblot analysis of pull down fractions. Homog, PC12 cell homogenate before loading onto Glutathione Sepharose; GST-MVI, a fraction eluted from the resin with attached MVI globular tail fused with GST, and GST, a fraction eluted from the GST-attached resin. The fractions were probed with antibodies against proteins listed on the panel that were identified by mass spec analysis (see Table 2). B, MVI (in green) colocalizes with hnRNPU (in red) in stimulation-dependent manner. Nuclear chromatin was visualized with ToPro3 dye (in blue). C, probing interaction of MVI with hnRNPU (left panel) and nucleolin (middle panel) by means of the PLA assay. Right panel, cells analyzed only with secondary antibodies (negative control). Bars, 10 μm. D, co-immunoprecipitation of endogenous hnRNPU with anti-MVI antibody (upper panel) or endogenous MVI with anti-hnRNPU antibody (lower panel) from stimulated PC12 cells. Cell homogenates (Homog), samples precipitated anti-MVI or anti-hnRNPU antibodies (α-IP) or normal rabbit serum (NS) were probed with anti-hnRNPU or anti-MVI antibodies as marked on the Figure. E, MVI (in red) colocalizes with nucleolin (in red) in stimulated cells. Bars in B and E, 5 μm. F, MVI (in green) colocalizes with histone 3 acetylated on lysine residue 9 (H3AcK9; in red) in stimulated cells. Bars in C and F, 10 μm. Nuclear chromatin in C, E and F was visualized with DAPI (in blue). Images in B, C, E and F represent confocal sections (0.3 μm) of the cell center.

As shown in Figure 4A and Supplementary Figure 5, MVI colocalized with SC35, and in stimulated cells the fluorescence intensity associated with SC35 was markedly increased and this was accompanied by an increase in its colocalization with MVI. However, colocalization of MVI with PML bodies was unaffected by cell stimulation (see Figure 4B and Supplementary Figure 6).

### MVI association with the proteins interacting with nucleic acids

Since MVI acts through interaction with its partners, we performed a search for proteins interacting with MVI in PC12 cells by means of a pull down assay. Affinity chromatography of PC12 cell homogenates on GST-fused MVI globular tail domain (GST-GT-MVI) bound to Glutathione-Sepharose was followed by detection of the bound proteins by tandem mass spectrometry. Out of 140 proteins identified in the sample eluted from the column with GST-GT-MVI attached to the resin *via* glutathione nearly a half (74 proteins) was not detected in the control sample (*i.e*. eluted from Glutathione Sepharose with GST alone). Only 35 proteins met our criteria, *i.e*. not only were present in the GST-GT-MVI sample and not in the control one but were also identified by at least three distinct peptide spectra with mass score value over 90 (Table 2). Interestingly, only five known MVI partners were detected [32,40–43] whereas the remaining 30 were potentially novel MVI partners.

**Table 2.** List of potential MVI-binding partners in PC12 cells identified by mass spectrometry

Gene ID	Human gene name	*Name*	Mass score	Peptide number
KNOWN PARTNERS				
gi|71362801	*DOCK7*	dedicator of cytokinesis 7 [32]	794	26
gi|212549641	*LMTK2*	lemur tyrosine kinase 2 [40]	173	3
gi|56605806	*TOM1*	target of myb1 homolog [41]	154	3
gi|4501885	*ACTB*	β-actin[42]	150	4
gi|13162357	*DAB2*	disabled homolog 2, mitogen-responsive phosphoprotein[43]	110	4
NOVEL PARTNERS Nuclear functions				
gi|41056215	*XRCC5*	X-ray repair complementing defective repair in Chinese hamster cells 5	653	18
gi|149060668	*LRCH3*	leucine-rich repeats and calponin homology (CH) domain containing 3 (predicted), isoform CRA_a	498	12
gi|18181882	*G22P1*	G22p1 – ATP-dependent DNA helicase activity	491	15
gi|197927118	*LRCH1*	leucine-rich repeats and calponin homology (CH) domain containing 1	346	12
gi|149065924	*DDX17*	DEAD (Asp-Glu-Ala-Asp) box polypeptide 17, isoform CRA_a	338	10
gi|149016333	*NCL*	nucleolin, isoform CRA_d	308	8
gi|157819755	*DDX3Y*	hypothetical protein LOC364073	302	9
gi|157823027	*DDX3X*	DEAD (Asp-Glu-Ala-Asp) box polypeptide 3, X-linked	285	8
gi|624918	*HNRNPU*	SP120 /heterogeneous nuclear ribonucleoprotein U	234	7
gi|109504268	*WRN*	PREDICTED: similar to Werner syndrome ATP-dependent helicase homolog	233	6
gi|149023880	*PABP family*	rCG31475, isoform CRA_c	230	5
gi|56090441	*DDX5*	DEAD (Asp-Glu-Ala-Asp) box polypeptide 5	205	8
gi|13384620	*HNRNPK*	heterogeneous nuclear ribonucleoprotein K	152	4
gi|204595	*HIST1H1D*	histone (H1d)	121	3
gi|114145419	*RPA1*	replication protein A1	96	3
gi|158186696	*HNRNPM*	heterogeneous nuclear ribonucleoprotein M isoform b	90	3
Ribosomal/ER functions				
gi|4506725	*RPS4X*	ribosomal protein S4, X-linked X isoform	335	7
gi|6677809	*RPS6*	ribosomal protein S6	318	5
gi|19705459	*PABPC1*	poly(A) binding protein, cytoplasmic 1	278	9
gi|129731	*P4HB*	RecName: Full = Protein disulfide-isomerase; Short = PDI	269	8
gi|11693176	*RPLP0*	acidic ribosomal phosphoprotein P0	201	5
gi|4506661	*RPL7A*	ribosomal protein L7a	168	4
gi|8394221	*RPS3A*	ribosomal protein S3a	140	7
gi|206736	*RPL7*	ribosomal protein L7	138	4
gi|2920825	*RPS2*	ribosomal protein S2	133	4
gi|114145636	*SYNCRIP*	synaptotagmin binding, cytoplasmic RNA interacting protein	111	3
gi|6755372	*RPS3*	ribosomal protein S3	95	4
Other functions				
gi|157823763	*TOLLIP*	Toll interacting protein (TOLLIP), innate immune system	235	7
gi|157817827	*DOCK6*	Dedicator of cytokinesis 6, guanyl-nucleotide exchange factor for Rac and Cdc42	214	7
gi|195540035	*TRIM41*	Trim41 protein, E3 Ubiquitin-Protein Ligase TRIM41	175	3

Most of these potential partners are proteins interacting with nucleic acids and/or involved in transcription and post-transcriptional processes such as maturation of newly formed transcripts, and nuclear export (Table 2). Among them are three heterogeneous nuclear ribonucleoproteins, hnRNPU, hnRNPK and hnRNPM, and histone H1d as well as nucleolin, a protein involved in nucleolar functions. The other big group of the putative MVI partners comprises ribosomal proteins, indicating possible involvement of MVI in protein synthesis. This is in line with our previous observation showing the presence of MVI in endoplasmic reticulum of PC12 cells [26] (see also Supplementary Figure 1d).

In order to verify these putative interactions, we performed immunoblot analysis of the pull down fractions with the use of antibodies against selected representatives of the MVI potential partners. As shown in Figure 5A, we detected hnRNPU and two other proteins of heterogeneous nuclear ribonucleoprotein complex [44], namely hnRNPL and hnRNPA2/B1. Moreover, nucleolin and ribosomal protein S6 were also detected. Noteworthy, interaction of MVI with S6 seems to be more specific for its active phosphorylated form (pS6), suggesting a functional relevance of this interaction. To verify the specificity of the interactions, we tested whether the PTBP1 [45], a member of the hnRNP complex, that was not identified in the course of the mass spec analysis could be immunodetected in the eluate. As demonstrated in Figure 5A, it was not found neither in the fraction eluted from the MVI-tail attached resin nor in the fraction eluted from GST-attached resin.

We next examined whether some of these interactions, namely with hnRNPU and nucleolin, could take place *in cellulo*. As shown in Figure 5B and Supplementary Figure 7, hnRNPU colocalized with MVI in a stimulation-dependent manner. This interaction was further confirmed by co-immunoprecipitation as both hnRNPU and MVI were present in the fractions precipitated from PC12 cell homogenates with anti-MVI or anti-hnRNPU antibodies (Figure 5D). It should be noted that though MVI co-localizes with both SC35 and PML, hnRNPU is not present in the regions stained with these two nuclear markers (Supplementary Figure 8A). We also employed the proximity ligation assay (PLA) that has been designed for *in situ* detection of two proteins existing within close intracellular proximity (within the 20–40 nm range), and the positive PLA signal (here in red) is considered as an evidence for their interaction [46]. The positive signals for hnRNPU-MVI visible in the nuclei and perinuclear region (Figure 5C) confirm interaction of these two proteins *in cellulo* thus suggesting its functional relevance. We also showed that in stimulated PC12 cells MVI co-localized with nucleolin (Figure 5E and Supplementary Figure 8B), and the PLA assay confirmed that both proteins could interact *in cellulo* (Figure 5C).

Additionally, since one of the histones, H1d, was identified as a putative MVI interaction partner, we examined whether MVI could localize to the active histone complex. Staining with the antibody directed against histone H3 acetylated on lysine residue (K9), generally accepted as a tool in studies on histone functions, revealed that in stimulated cells MVI colocalized with the active form of this protein (Figure 5F). This observation suggests that MVI could indeed interact with histone complexes during transcription.

## Discussion

Our study demonstrates for the first time a stimulation-dependent increase of MVI in the nucleus that is associated with its localization within transcription sites. We have also found several novel MVI binding partners, predominantly the ones involved in nuclear/ribosomal processes and confirmed MVI interaction *in cellulo* with two of them, hnRNPU and nucleolin, proteins involved in pre-mRNA metabolism and transport, and nucleolar processes, respectively. We hypothesize that interactions with these newly identified partners could be responsible for engagement of MVI in nuclear processes and its shuttling between the cytoplasm and the nucleus.

Several reports demonstrated involvement of MVI in cell proliferation and nuclear processes, especially in cancer cells [23–30]. For example, elevated expression of MVI was linked with aggressive phenotypes of ovarian and prostate cancers in humans and mice [23,24] and association of MVI with active RNAPII was shown for HeLa cells [27]. Moreover, our observations that MVI is present within the nuclei of primary bovine adrenal medulla chromaffin cells, skeletal muscle, highly motile unicellular *Amoeba proteus* and developing mouse spermatids [16,26,47,48] indicate that MVI in the nuclei is not only associated with pathology but may also have physiological role(s) in non-transformed cells/tissues.

The PC12 cell line was derived from rat pheochromocytoma, adrenal medulla tumor, and is a widely accepted model of neurosecretory cells [49]. Physiological activities of PC12 cells are associated with catecholamine secretion and therefore are accompanied by exceptionally high turnover of membranes and various particles, requiring constitutive and fast rebuilding of their resources. Hence, the processes associated with transcription and protein synthesis are greatly enhanced there, suggesting that there might be a need for elevated amounts of motor proteins such as for example MVI. It is plausible that MVI could facilitate the nuclear activities by transporting the substrates and products into and out the nucleus, and/or similarly to NMIC be engaged directly in transcription processes by interacting with complexes of RNA polymerases.

The presence of several potential NLS sequences and a potential NES signal within MVI heavy chain, originally indicated by Vreugde et al. (2006) [27], implies engagement of nuclear pore complex-associated transporting machinery, and nuclear-cytoplasmic shuttling of this molecular motor in response to cell stimuli. We postulate that a strong nuclear phenotype observed for MVI in PC12 cells could be explained by the fact that a predominant MVI isoform is the one lacking a large insert that spatially blocks the region of the cargo domain with the bipartite NLS [36]. A change in nuclear localization of MVI evoked by inhibitors of nuclear import and export seems to confirm this speculation. Noteworthy, nuclear localization of myosin Va (MVA) is regulated by phosphorylation of its S1650 residue [50], whereas nuclear localization of NMIC, initially thought to depend on the presence of the highly conserved positively charged N-terminal 16-amino-acid sequence, is probably associated with second IQ motif serving as calmodulin-dependent non-canonical NLS sequence [51–53]. In this respect, it is interesting that one of the MVI putative NLS sequences is located within the IQ region (see Table 1). Nuclear translocation of myosin XVIIIB into the nucleus associated with muscle differentiation was also reported [54].

Immunostainings along with FRAP and time-lapse experiments demonstrated not only MVI translocation to the nucleus but also its mobility within the nucleus that seems to be required for postulated MVI functions such as involvement in transcription and posttranscriptional events. The grounds for this postulation come from the present study *i.e*. colocalization of MVI with several constituents of transcription machinery and within decondensed chromatin as well as with PML bodies, and from our previous report demonstrating important role for MVI in cell proliferation [31]. Of note, it is interesting why colocalization with PML bodies does not depend on cell stimulation. It should be recalled that other groups showed involvement of MVI in RNAPII activity, p53-dependent DNA damage and chromosome pairing [27,28,30].

The question arises as of what could be the mechanism(s) of MVI involvement in transcription and MVI-based trafficking of particles to and from as well as within the nucleus. So far, more detailed data addressing myosin functions in the nucleus are available only for NMIC [1,3]. It was demonstrated that NMIC could bind to transcription factors (for example TIF1A) and interact with RNAPI *via* actin. Moreover, the presence of NMIC functional motor domain is indispensable for initiation of transcription thus suggesting that ATP-dependent interaction of this myosin with actin takes place also in the nucleus[50,55–57]. Other studies suggest that NMIC is also involved in a long-range translocation of interphase chromosomes after transcription initiation [58]. Likewise, it was demonstrated that NMIC might be involved in chromatin remodeling through its interaction with WSTF and SNF2h members of the remodeling complex [59].

As it was mentioned earlier, MVI fulfills its functions through binding to actin, also present in the nuclei, and to its tissue- and cell type-specific partners interacting with C-terminal globular tail domain of MVI [8–12]. Our search for MVI partners resulted in identification of several novel putative partners, and a majority of them is implicated in interactions with nucleic acids and in transcript maturation and trafficking (see Table 2). In line with this finding is a report describing interaction of murine MVI with TLS (Translocated in Liposarcoma), a protein involved in rapid nuclear-cytoplasmic shuttling of spliced mRNA in hippocampus [60]. Also, it was shown that in the budding yeasts Myo2p (one of two myosin V isoforms in *S. cerevisiae*) was associated with a large ribonucleic acid-protein complex containing mRNAs and subunits of the RNA-processing entity [61]. Moreover, it was reported that MVI was recruited to promoter and intragenic regions of several active genes thus affecting their transcription [27]. Therefore, it is plausible that MVI could be recruited to DNA (and RNA) *via* DNA(RNA)-binding proteins and that interaction with partners taking place in a particular nuclear region determines its specific function. For example, one could expect that interaction of MVI with nucleolin is important for nucleolar functions, interaction with heterogeneous ribonucleoproteins suggests involvement of MVI in RNA maturation and transport, interaction with histones implies MVI role in chromatin organization, and interaction with S6 ribosomal protein suggests engagement of MVI in translation. Interestingly, a recent study [62] showed that expression of the MVI gene in hnRNPA1-defect mice was significantly increased, confirming significance of the MVI interaction with subunits of the RNP complex. Our data confirming *in cellulo* interaction between MVI and hnRNPU indicate functional relevance of this interaction as well. Noteworthy, we identified hnRNPU and S6 as putative MVI partners also in skeletal muscle [16]. Further studies are required to characterize these novel partnerships and their regulation. For example, it is not clear whether the identified interactions reflect direct or indirect binding as the identified proteins and MVI could be in the same complexes and not simply bound to each other. Mapping of the interaction sites performed on purified recombinant proteins or their fragments and possibly mutants will be certainly helpful.

Summarizing, our study indicates that MVI could be involved in nuclear processes and nuclear transport through its interaction(s) with the proteins involved in gene expression.

## Materials and methods

### Plasmids and reagents

The GFP-MVI construct (widely used to overexpress fluorescently tagged MVI) encoding the human MVI heavy chain cDNA [63] (gene ID Q9UM540, isoform without a large insert, predominant in PC12 cells [26]) was from Dr. Tama Hasson from University of California, Los Angeles. Plasmid for expression of recombinant globular tail domain of MVI fused with GST (glutathione S-transferase) in *E. coli* was constructed by subcloning the fragment of rat MVI nucleotide sequence [32] (gene ID D4A5I9) corresponding to the MVI globular tail (aa 1046–1285) into pGEX-4T1 vector (from GE Healthcare, Cat No 28-9545-49). Glutathione Sepharose 4B was also from GE Healthcare (Cat. No 17-0756-01). Cell culture reagents were from Invitrogen and ATCC. Leptomycin B was from Sigma-Aldrich (Cat. No L2913) and ivermectin was from Sigma-Aldrich (Cat. No I8898). Ten percent acrylamide precast Tris-HCl Ready Gels were from Bio-Rad Laboratories (Cat. No 1611155). Sequencing Grade Modified Trypsin was from Promega (Cat. No V5111) and 0.1% formic acid solutions in water and acetonitrile were from Avantor Performance Materials (Cat. No 9834-02 and 10264011, respectively). All other reagents were from Sigma-Aldrich.

### Antibodies and fluorescent markers

Rabbit polyclonal antibody directed against amino-acid residues 1049–1054 of porcine MVI heavy chain, originally developed by Dr. Tama Hasson was from Proteus (Cat. No 25–6791) and rabbit anti-hnRNPU polyclonal antibody was from Proteintech Europe (Cat No 14599–1-AP). Following monoclonal antibodies were used: anti-lamin B, anti-SP1, anti-hnRNPU, anti-nucleolin and anti-histone H3 acetylated at lysine residue 9 (AH3-120) were from Santa Cruz Biotechnology (Cat. No sc-6217, sc-59, sc-32315, sc-8031 and sc-56616); anti-PML and anti-GAPDH were from Millipore (Cat. No MAB3738 and MAB374, respectively); anti-SC35, anti-RNAP II, anti-BrdU anti-hnRNPL and anti-hnRNPA2/B1 were from Abcam (Cat. No ab11826, ab24759, ab1893, ab6106 and ab6102, respectively); anti-S6 and anti-pS6 were from Cell Signalling (Cat. No 2217S and 4858S, respectively). ToPro3 was from Invitrogen (Cat. No T3605) and DAPI was from Vector Labs (Cat. No H-1200). For immunofluorescence studies, the following secondary antibodies were used: goat anti-rabbit IgG labeled with Alexa Fluor 488 and goat anti-mouse IgG labeled with Alexa Fluor 546 (both from Invitrogen, Cat. No A11008 and A11003, respectively). *In situ* proximity ligation assay (PLA) kit was from Sigma-Aldrich (kit components: Duolink InSitu PLA probe anti-mouse minus, Cat. No DUO 92004, Duolink InSitu PLA probe anti-rabbit plus, Cat. No DUO 92002, Duolink in situ detection reagents red Cat. No DUO 92008, Duolink in situ wash buffers, Cat. No DUO 82049, Duolink in situ mounting medium with DAPI, Cat. No DUO 92006).

### Cell culture and fractionation

PC12 cells (from American Cell Culture Collection, ATCC, Cat. No CRL-1721) were cultured in RPMI media (Gibco, Cat. No 52400025) supplemented with 10% heat-inactivated horse serum (Gibco, Cat. No 26050088), 5% heat inactivated fetal bovine serum (Gibco, Cat. No 10270106) and antibiotics: penicilin/streptomycin (100 μg/ml; Gibco, Cat. No 15140–122) at 37°C under 5% CO2, according to [47]. Cells were lysed in an ice-cold buffer that contained 50 mM Tris–HCl pH 8.0; 150 mM NaCl, 0.1% Triton X-100, 2 mM EGTA, 1 mM DTT, 1 mM PMSF and Complete protease inhibitor cocktail (Roche, Cat No. 04693116001). For fractionation, cell homogenates (t) were spun for 10 min at 2700 x g and the resulted supernatants (cytoplasmic fraction, s) and pellets (crude nuclear fraction, p) were subjected to the SDS-PAGE followed by the immunoblot analysis for the presence of MVI and other marker proteins (GAPDH for the cytoplasm and lamin B for the nucleoplasm) that were used as the internal load control and fraction purity using the relevant antibodies. Protein concentration was determined using the standard Bradford method.

### Transfection with GFP-MVI constructs

PC12 cells grown in six-well dishes were transfected with 2 μg/well of purified GFP-MVI plasmid using Lipofectamine 2000 (Invitrogen, Cat. No 11668027) according to manufacturer's instructions. Cells were incubated with transfection reagent for 16–20 h to obtain adequate MVI expression levels.

### Cell stimulation

To induce secretion, PC12 cells were cultured as described above and stimulated essentially according to [64,65]. High concentrations of external KCl (56 mM) cause the PC12 cell plasma membrane depolarization and evoke catecholamine release. Therefore treatment with KCl is generally accepted as a way of in vitro PC12 cell stimulation. Cells were washed with Locke's solution that contained 2.6 mM KCl, 154 mM NaCl, 2.2 mM CaCl_2_, 0.5 mM KH_2_PO4, 1.25 mM K_2_HPO_4_, 1.2 mM MgCl_2_ and 10 mM glucose. Then, they were incubated in Locke's solution with elevated K^+^ concentration (56 mM KCl, 103.6 mM NaCl, 2.2 mM CaCl_2_, 0.5 mM KH_2_PO_4_, 1.25 mM K_2_HPO_4_, 1.2 mM MgCl_2_, 10 mM glucose) to stimulate the secretion, or in calcium-free Locke's solution (154 mM NaCl, 2.6 mM KCl, 0.5 mM KH_2_PO_4_, 1.25 mM K_2_HPO_4_, 1.2 mM MgCl_2_ and 10 mM glucose) to block the secretion. The cells were further processed for immunocytochemistry or fractionation. *In vivo* visualization of translocation of the GFP-MVI to the nucleus was performed using a time-lapse technique. The cells 24 hours after transfection were filmed with 15-s time lapse using Leica TCSP5 confocal microscope equipped with a temperature-controlled live-cell imaging chamber.

### Leptomycin B and ivermectin treatment

PC12 cells were plated on poly-D-lysine-coated glass coverslips and grown overnight as described above. Leptomycin B (20 nM) was used to arrest the nuclear export and ivermectin (25 μM) to block the nuclear import of proteins. Cells were first treated with the inhibitors for 4 hours and after 5-min stimulation were subjected to immunofluorescence staining for MVI.

### Fluorescence Recovery after Photobleaching (FRAP)

GFP-MVI was expressed in a variant of PC12 cells (ATCC, Cat. No CRL-1721.1) as described above. These variant cells are not responding to stimulating factors (such as KCl or NGF) but are better attached to the surface. FRAP was carried out using Leica TCS SP5 laser confocal microscope with HCX PL APO 63×/1.25-0.75 Oil Cs objective. GFP fluorescence was excited using the 488 nm line of the Ar ion laser and emission was collected between 500 and 560 nm at a 1 Airy disc pinhole size. Pixel size was 122 nm × 122 nm. Scanning was performed at 400 – 800 Hz with laser power set to 35% (gain 720V; offset 0.5-3%). Bleaching was carried out with laser power set to 100%. FRAP experiments were performed and analyzed using the FRAP-wizard tool of the Leica Confocal Software Version 2.6.1 Build 1537 (Leica Microsystems). In brief, recordings started by taking 5 frames at maximal speed (2.93 s/frame). Then a 7.44 nm × 7.44 nm region of interest (ROI) was bleached three times (using the “zoom in” option), and 10 frames were recorded at maximal speed followed by taking 5 frames at 5-s interval. For calculating the half-time of recovery, the resulting curves were fitted to a single exponential function (using FRAP-wizard tool). Signals in the ROI were background corrected. Half times of recovery were obtained from three independent experiments. Images were exported into Adobe Photoshop.

### Immunoblotting

PC12 cell fractions were separated using 10% polyacrylamide SDS gels and then transferred to a nitrocellulose membrane (BioRad, Cat. No 1620115). After the transfer, the membrane was blocked for 1 h at room temperature in TBS containing 5% non-fat milk powder and 0.2% Triton X-100 followed by 1-hour incubation with appropriate dilutions (from 1:200 to 1:500) of different antibodies described above. The primary antibodies were detected using a 1:10000 dilutions of anti-rabbit (Millipore, Cat. No AP307P), anti-mouse (Millipore, Cat. No AP308P) or anti-donkey (Santa Cruz Biotechnology, Cat No sc-2020) secondary antibodies conjugated with horse radish peroxidase; the reaction was developed using the ECL detection kit (Pierce, Cat. No 34095). Usually 10–20 μg of protein was loaded onto the gel. The content of MVI in particular fractions was estimated by densitometric analysis of immunoblots that were photographed using G:Box system from SynGene (Cambridge) equipped with Gene Snap and GeneTools software.

### Immunolocalization studies

Distribution of MVI and other examined proteins in PC12 cells was evaluated before and after stimulation with 56 mM KCl by indirect immunocytochemistry. Cells on coverslips were fixed in 4% paraformaldehyde for 30 min, washed three times with phosphate-buffered saline (PBS) and blocked in solution that contained 2% horse serum, 0,02% Triton X-100 in PBS for 1h at room temperature. Coverslips were then incubated overnight at 4^o^C with anti MVI antibody (at 1:50 dilution) and washed three times in PBS with 0,02% Triton X-100. This was followed by incubation with Alexa 488-conjugated anti-rabbit secondary antibody at a dilution of 1:1000 for 60 min. For simultaneous assessment of distribution of other proteins, cells were also incubated with the antibodies above described (at 1:50 dilutions), and then with Alexa 546-conjugated secondary anti-mouse antibody (1:1000 for 60 min). ToPro3 or DAPI stainings were performed according to the manufacturer's instructions. The specimens were visualized using Leica TCS SP2 or TCS SP5 spectral confocal microscopes equipped with HCX PL APO 63x/1.25-0.75 Oil Cs objective, or with Zeiss LSM780 spectral confocal microscope equipped with a Plan-Apochromat 63x/1.40 Oil DIC M27 lens. In double immunostainings, special care was taken to control for any possible cross-reactivity (cross-bleeding) of the detection systems. We carefully adjusted the spectral ranges of detectors and always scanned the images sequentially. For negative controls, the primary antibody was omitted. Highest-resolution confocal images were restored by three dimensional (3D) deconvolution using Huygens Professional software (Scientific Volume Imaging, Hilversum, Netherlands, http://www.svi.nl/) by applying classic maximum-likelihood estimation algorithm and automatically-generated point-spread function. The deconvolved data sets were then imported into Image Pro Plus 6.3 Software (Media Cybernetics Inc., Bethesda MD) for colocalization analysis. A quantitative assessment of fluorophore colocalization in confocal optical sections was performed using Pearson's correlation coefficient, which is a well defined and commonly accepted tool for describing the extent of overlap between image pairs [66]. As a result, the value of this coefficient ranges from -1 to 1, with a value of -1 representing a total lack of overlap between pixels from the images, and a value of 1 indicating perfect image registration. Area of interest (AOI) within the scatterplot marks the colocalized areas and the resulted mask reflects overlapping regions of red and green channels. To determine the amount of fluorescent signals in nucleus and cytoplasm associated with MVI, the following analysis was performed using Leica Software (Leica); in total more than 100 confocal images were tested. To estimate the levels of fluorescence, a function measuring the frequency of the intensity was used. Statistical analysis was made using Statistica program (Statistica Software, Statsoft Inc.) and statistical significance was determined by Student's t-test and values of *p* ≤ 0.05 were considered significant.

### Post-embedding immunogold localization of MVI

PC12 cells were grown on Thermanox™ coverslips (Electron Microscopy Sciences, Cat. No 72274) as described earlier. The cells were gently rinsed with PBS and fixed with 4% (v/v) formaldehyde and 0.25% (v/v) glutaraldehyde in the same PBS buffer for 1 hour at room temperature. Fixed cells were washed three times with PBS, dehydrated in graduated ethanol concentrations, and embedded in LR White resin (Electron Microscopy Sciences, Cat. No 14380) according to the standard protocol. Ultrathin sections were cut using a diamond knife (Micro Star Technologies) and a Leica UTC ultramicrotome, and collected on Formvar-film coated nickel grids (Electron Microscopy Sciences, Cat. No FCF400-Ni). The sections were then pre-treated with 50 mM glycine in PBS for 10 min and incubated with blocking solution containing 3% (w/v) bovine serum albumin (BSA) in PBS for 5 min at room temperature. Next, sections were kept in 1:50 dilution of a primary MVI antibody in PBS supplemented with 0.3% BSA for 2 h, followed by incubation with a gold-conjugated anti-rabbit IgG 15-nm secondary antibody (BB International, Cat. No R14003) at 1:100 dilution in PBS with 0.1% BSA for 30 min. Both incubations were proceeded at room temperature. In the negative control, the primary antibody was omitted. Finally, the sections were stained with 2.5% uranyl acetate and examined on a Joel EM 1010 transmission electron microscope. Over 20 images of unstimulated and stimulated cells were used for a quantitative analysis.

### BrUTP incorporation assay

After storage at 4°C in 70% ethanol, the coverslips with fixed (stimulated and unstimulated) cells were rinsed with PBS, treated for 15 min with 0.5% Triton X-100 in PBS with shaking at room temperature, then rinsed with PBS. For cells that incorporated BrUTP, diethyl pyrocarbonate-treated PBS was used for all steps of the staining procedure. The coverslips were incubated in 1% Blocking Reagent (Roche) in PBS with 0.02% Tween 20 for 30 min at 37°C. Antibodies were diluted with 0.5% Blocking Reagent solution with 0.02% Tween 20. The incubations were performed at 37°C, and between subsequent incubations the slides were washed by shaking for 30 min in PBS supplemented with 0.1% Tween 20. The triple staining for incorporated BrUTP, MVI and ToPro3 was next performed. Cells were incubated with anti-BrDU antibody (1:100) followed by incubation with secondary anti-mouse antibody conjugated with Alexa Fluor 546, and then with polyclonal rabbit anti-MVI antibody (1:50) followed by incubation with secondary anti-rabbit antibody conjugated with Alexa Fluor 488 and ToPro3.

### GST pull-down assay and mass spectrometry analysis

The fusion protein composed of GST and MVI C-terminal globular tail domain (GST-MVI-GD) as well as GST alone were purified as described by [32]. Cells were lysed in ice-cold buffer that contained 50 mM Tris (pH 7.5), 150 mM NaCl, 0.5% Triton X-100, 1 mM DTT, 5 mM EGTA, 50 mM NaF, 1 mM Na_3_VO_4_, 0.5 mM PMSF and supplemented with the Complete protease inhibitor cocktail. The assay was performed as described in [32]. Briefly, homogenates were pre-cleared with GST-bound Glutathione Sepharose 4B beads for 2 hours at 4°C to remove proteins non-specifically binding to Glutathione Sepharose 4B or to GST, and subsequently incubated with Glutathione Sepharose 4B beads bound with GST-MVI fusion protein or GST alone. The beads were exhaustively washed in the ice-cold buffer described above and subjected to SDS–PAGE electrophoresis. Equal gel pieces were excised from the experiment (GST-fusion MVI globular tail) and control (GST alone) gel lanes. Prior to the analysis excised gel slices were subjected to the standard procedure of in-gel trypsin digestion, during which proteins were reduced with 100 mM DTT for 30 min at 56°C, alkylated with iodoacetamide in darkness for 45 min at room temperature, and digested overnight with 10 ng/μl trypsin. Peptides were eluted from gel with 0.1% formic acid (FA) and 2% acetonitrile (ACN), and were applied to RP-18 pre-column (Waters, Cat. No 186003977) using 0.1% FA water solution as a mobile phase and then transferred to a nano-HPLC RP-18 column (internal diameter 75 μm) using ACN gradient (0–30% in 45 min) in the presence of 0.1% FA at a flow rate of 250 nl/min. The column outlet was coupled directly to the ion source of LTQ FTICR mass spectrometer (Thermo Electron Corp., Cat. No 13437) working in the regime of data-dependent MS to MS/MS switch. A blank run ensuring absence of cross-contamination from previous samples preceded each analysis. After pre-processing the raw data with Mascot Distiller software (version 2.1.1, Matrix Science), obtained peak lists were used to search the non-redundant protein database of the National Centre for Biotechnology Information (NCBI) version 20090922 (9738651 sequences, 68328 Rattus sequences) using the Mascot search engine (version 2.2.03, 8-processors onsite license; Matrix Science) with the following search parameters: taxonomy restriction – Rattus norvegicus (rat), enzyme specificity – semi-trypsin, permitted number of missed cleavages – 1, fixed modification – carbamidomethylation (C), variable modifications – carbamidomethyl- ation (K) and oxidation (M), protein mass – unrestricted, peptide mass tolerance – ± 40 ppm, fragment mass tolerance – ± 0.8 Da, max missed cleavages – 1. Only proteins for which at least three peptides with Mascot cut-off scores ≥ 90, indicating identity or extensive homology of a peptide (p ≤ 0.05) identified in GST-MVI tail sample and excluding peptides identified in GST alone sample were considered as the positive identifications. Mass spectrometry analysis was performed in the Institute of Biochemistry and Biophysics Polish Academy of Sciences, Warsaw, Poland

### Proximity ligation assay (PLA)

PC12 cells after fixation were blocked in Duolink blocking solution in a humidity chamber for 30 min at 37°C, and incubated with primary antibodies: rabbit polyclonal anti-MVI (1:50), and mouse monoclonal against hnRNPU or nucleolin (both at 1:50) in Duolink Antibody diluent solution for 3h at 37°C. Cells were next washed twice in a wash buffer for 5 min at room temperature. For secondary antibodies conjugated with oligonucleotides, PLA probe anti-mouse MINUS and PLA probe anti-rabbit PLUS were applied in Duolink antibody diluent solution for 1h at 37°C, and washed twice with for 5 min. Duolink assay was further performed strictly according to the manufacturer's instructions. For negative controls, the primary antibodies were omitted.

### Co-immunoprecipitation

To perform co-immunoprecipitation, PC12 cells were lysed in a buffer that contained 50 mM Tris (pH 7.5), 150 mM NaCl, 2 mM EDTA, 0.2% Triton X-100, 2 mM MgCl_2_, 2 mM MgATP, 50 mM NaF, 1 mM Na_3_VO_4_ and supplemented with the Complete protease inhibitor cocktail. The lysates were pre-cleared with A/G agarose beads (Santa Cruz Biotechnology, Cat. No sc-2003) for 30 min at 4°C and subsequently incubated for 1 h at 4°C with 10 µg of the anti-MVI antibody or 8 µg anti-hnRNPU antibody (or normal rabbit serum as a control) followed by overnight incubation of the mixture with the aforementioned agarose beads. The beads were washed with the lysis buffer and then subjected to the SDS–PAGE electrophoresis. Western blot was performed using anti-hnRNPU (1:50) or anti-MVI (1:500) antibodies to detect the co-immunoprecipitated MVI-hnRNPU complexes.

## Supplementary Material

supp_data_1421881.zip
